# Long-term oncologic outcomes of pulmonary metastasectomy for lung-confined recurrent endometrial cancer: a single-institution case series

**DOI:** 10.1016/j.xagr.2026.100705

**Published:** 2026-10-07

**Authors:** Minami Kawanaka, Akiho Nishimura, Takeshi Motohara

**Affiliations:** Department of Obstetrics and Gynecology, Faculty of Life Sciences, Kumamoto University, Kumamoto City, Kumamoto, Japan

**Keywords:** endometrial cancer, long-term survival, lung-confined recurrence, pulmonary metastasectomy, pulmonary metastasis

## Abstract

**Background:**

Pulmonary recurrence in endometrial cancer is generally regarded as a manifestation of systemic disease and is primarily managed with systemic therapy. However, the clinical significance of lung-confined recurrent endometrial cancer and the role of pulmonary metastasectomy remain uncertain.

**Objective:**

To evaluate the long-term oncologic outcomes of pulmonary metastasectomy in patients with lung-confined recurrent endometrial cancer and to explore whether lung-confined recurrence represents a clinically distinct pattern of recurrence.

**Study Design:**

This retrospective single-institution case series included consecutive patients with lung-confined recurrent endometrial cancer who underwent pulmonary metastasectomy between January 2005 and July 2026. Clinicopathological characteristics, long-term oncologic outcomes, and perioperative outcomes were evaluated.

**Results:**

Six patients underwent pulmonary metastasectomy for lung-confined recurrent endometrial cancer. The median interval from primary surgery to first pulmonary recurrence was 45.5 months (range, 19–227 months). Complete (R0) pulmonary resection was achieved in all patients without severe surgery-related adverse events. During a median follow-up of 103.5 months (range, 47–250 months) from primary surgery, all 6 patients remained alive, and 5 of 6 achieved durable disease-free status. Following the first pulmonary metastasectomy, 1 patient developed subsequent lung-confined recurrence and underwent repeat pulmonary metastasectomy, achieving additional long-term disease control. Notably, the 2 patients with synchronous bilateral pulmonary metastases also achieved durable disease-free survival following complete staged bilateral resection.

**Conclusion:**

Pulmonary metastasectomy may provide durable long-term disease control in carefully selected patients with lung-confined recurrent endometrial cancer. Recognition of lung-confined recurrent endometrial cancer as a clinically distinct pattern of recurrence may support a shift from routine systemic therapy alone toward a more individualized multidisciplinary treatment strategy incorporating pulmonary metastasectomy.

**AJOG Global Reports at a Glance**

**Why was this study conducted**

To evaluate the long-term oncologic outcomes of pulmonary metastasectomy in patients with lung-confined recurrent endometrial cancer and to determine whether this rare recurrence pattern represents a clinically distinct subset rather than conventional disseminated systemic disease.

**Key findings**

All 6 patients remained alive after pulmonary metastasectomy, 5 of 6 achieved durable disease-free status, and 1 patient with subsequent lung-confined recurrence underwent repeat pulmonary metastasectomy and achieved additional long-term disease control.

**What does this add to what is known?**

This study provides one of the longest reported follow-up series of pulmonary metastasectomy for lung-confined recurrent endometrial cancer and supports recognition of this recurrence pattern as a biologically and clinically distinct subset that may benefit from individualized multidisciplinary management incorporating surgery.

## Introduction

Endometrial cancer is the most common gynecologic malignancy in developed countries, and its incidence continues to increase worldwide.^1^^,^^2^ Although most patients are diagnosed with early-stage disease and achieve favorable outcomes after primary treatment, approximately 10% to 20% experience disease recurrence.^3^^,^^4^ Recurrent endometrial cancer is generally regarded as a manifestation of systemic disease, and distant recurrence is primarily managed with systemic therapy, including chemotherapy, immune checkpoint inhibitors, and molecularly targeted agents.^1^^,^^5^^,^^6^ Among distant recurrences, pulmonary recurrence is the most common site of metastasis and is generally considered indicative of disseminated disease rather than a potentially curable condition.^4^^,^^7^

However, clinical experience suggests that pulmonary recurrence is not a biologically uniform entity. A subset of patients develops pulmonary recurrence confined to the lungs without evidence of extrapulmonary dissemination. Patients with lung-confined recurrence have substantially more favorable outcomes than those with concomitant extrapulmonary disease.^7^, ^8^, ^9^ These observations suggest that lung-confined recurrence may represent a clinically distinct subset rather than invariably reflecting widespread systemic dissemination. When feasible, complete resection of pulmonary metastases may provide durable disease control.^8^, ^9^, ^10^ Nevertheless, the clinical significance of lung-confined recurrent endometrial cancer remains poorly understood, and the role of pulmonary metastasectomy has yet to be clearly defined.^4^^,^^7^^,^^8^^,^^11^

Pulmonary metastasectomy has been associated with favorable survival outcomes in carefully selected patients across several solid malignancies and has also shown promising results in recurrent gynecologic cancers.^8^, ^9^, ^10^^,^^12^ However, previous studies have generally included heterogeneous patient populations with variable recurrence patterns, limited follow-up periods, or limited numbers of patients with lung-confined recurrence. Consequently, the specific role of pulmonary metastasectomy in patients with lung-confined recurrent endometrial cancer remains uncertain.^4^^,^^8^

The aim of the present study was to evaluate the long-term oncologic outcomes of pulmonary metastasectomy in patients with lung-confined recurrent endometrial cancer treated at a single institution, with follow-up extending beyond 20 years in the longest-observed patient. We also investigated whether lung-confined recurrent endometrial cancer represents a clinically distinct pattern of recurrence that may warrant incorporation of pulmonary metastasectomy into an individualized multidisciplinary management strategy.

## Materials and methods

### Study design

This retrospective single-center case series was conducted at Kumamoto University Hospital. Consecutive patients with histologically confirmed endometrial cancer who underwent pulmonary metastasectomy for lung-confined recurrence between January 2005 and July 2026 were identified from the institutional database.

At the time of primary diagnosis, tumor stage was assigned according to the 2008 International Federation of Gynecology and Obstetrics (FIGO) staging system,^13^ and histological diagnosis was established based on surgical specimens according to the WHO Classification of Tumours: Female Genital Tumours (fifth edition, 2020).^14^

The study was approved by the Institutional Review Board of Kumamoto University Hospital (Approval No. 3423). Written informed consent was obtained from all patients in accordance with institutional guidelines.

### Patient selection

Patients were eligible if they met all of the following criteria: (1) histologically confirmed endometrial cancer treated with curative-intent primary surgery; (2) lung-confined recurrence without radiological evidence of extrapulmonary disease; and (3) pulmonary metastases considered amenable to complete resection following multidisciplinary evaluation. Patients with diffuse or unresectable pulmonary metastases were excluded.

### Preoperative evaluation for pulmonary metastasectomy

All patients underwent contrast-enhanced computed tomography and/or positron emission tomography-computed tomography before pulmonary metastasectomy to confirm the absence of extrapulmonary disease. Surgical eligibility was determined through multidisciplinary review involving gynecologic oncologists, thoracic surgeons, radiologists, and pathologists, based on the extent of pulmonary disease, technical resectability, and the absence of extrapulmonary metastases.

### Pulmonary metastasectomy

Pulmonary metastasectomy was performed thoracoscopically (video-assisted thoracoscopic surgery [VATS]) in all patients by experienced thoracic surgeons with curative intent. The extent of pulmonary resection (wedge resection, segmentectomy, or lobectomy) was determined according to the size, number, and anatomical location of the metastatic lesions while aiming to achieve complete (R0) resection with maximal preservation of pulmonary function. Lymph node dissection was not routinely performed; however, 1 patient underwent lymph node dissection. Postoperative adverse events were graded according to the Common Terminology Criteria for Adverse Events (CTCAE) version 5.0.

### Outcome measures

The study outcomes included overall survival (OS), progression-free survival (PFS), postmetastasectomy progression-free survival (PM-PFS), postrecurrence survival (PRS), perioperative outcomes, postoperative morbidity, and disease status at the last follow-up. OS was defined as the interval from primary surgery to death from any cause, whereas PFS was defined as the interval from primary surgery to the first documented disease recurrence or death from any cause. PM-PFS was calculated from the date of the first pulmonary metastasectomy to disease progression, recurrence, or death, whereas PRS was calculated from the date of the first pulmonary recurrence to death from any cause.

### Statistical analysis

Continuous variables are presented as the median (range), and categorical variables as frequencies and percentages. Survival outcomes, including median PFS and median PM-PFS, were estimated using the Kaplan–Meier method. Statistical analyses were conducted using GraphPad Prism version 11.0 (GraphPad Software, San Diego, CA).

## Results

### Patient characteristics

Baseline clinicopathological characteristics are summarized in Table 1. Six patients with lung-confined recurrent endometrial cancer who underwent pulmonary metastasectomy were included in this study. The median age at the initial diagnosis of endometrial cancer was 60 years (range, 51–73 years). FIGO stage was IA in 2 patients, IB in 2, IIIA in 1, and IIIC1 in 1. Histological subtypes included grade 1 endometrioid carcinoma (n=2), grade 3 endometrioid carcinoma (n=1), carcinosarcoma (n=2), and mesonephric-like adenocarcinoma (n=1). All patients underwent primary gynecologic surgery, consisting of total abdominal hysterectomy (n=2) or modified radical hysterectomy (n=4). Pelvic lymphadenectomy was performed in 5 patients. Four patients had lymphovascular space invasion, and pelvic lymph node metastasis was identified in 1 patient. Four patients received adjuvant cytotoxic chemotherapy based on pathological risk factors.

**Table 1:** Baseline clinicopathological characteristics of patients with lung-confined recurrent endometrial cancer

Case	Age, years	Gravidity	Parity	BMI, kg/m²	Stage (FIGO 2008)	Histological type	Primary surgery	Myometrial invasion	LVSI	Lymph node metastasis	Adjuvant therapy
1	51	4	2	21.7	IA	Endometrioid carcinoma, Grade 1	AT+BSO	21%	No	No	No
2	73	3	2	24.7	IA	Carcinosarcoma	mRH+BSO+PLND+OMT	7%	No	No	TC (6 cycles)
3	61	3	3	23.5	IB	Mesonephric-like adenocarcinoma	AT+BSO+PLND	52%	Yes	No	No
4	65	2	2	22.5	IB	Carcinosarcoma	mRH+BSO+PLND+OMT	86%	Yes	No	TC (6 cycles)
5	59	0	0	25.7	IIIA	Endometrioid carcinoma, Grade 1	mRH+BSO+PLND+OMT	82%	Yes	No	TC (5 cycles)
6	58	3	1	24.3	IIIC1	Endometrioid carcinoma, Grade 3	mRH+BSO+PLND+OMT	88%	Yes	Yes	AP (6 cycles)

*AP*, doxorubicin plus cisplatin; *AT*, abdominal hysterectomy; *BMI*, body mass index; *BSO*, bilateral salpingo-oophorectomy; *LVSI*, lymphovascular space invasion; *mRH*, modified radical hysterectomy; *OMT*, omentectomy; *PLND*, pelvic lymph node dissection; *TC*, paclitaxel plus carboplatin.

### Clinical course

The clinical course of each patient is illustrated in Figure 1. After primary gynecologic treatment, all patients developed lung-confined recurrence following a disease-free interval without evidence of extrapulmonary disease. Pulmonary metastasectomy was subsequently performed in all patients.

**Figure 1:**
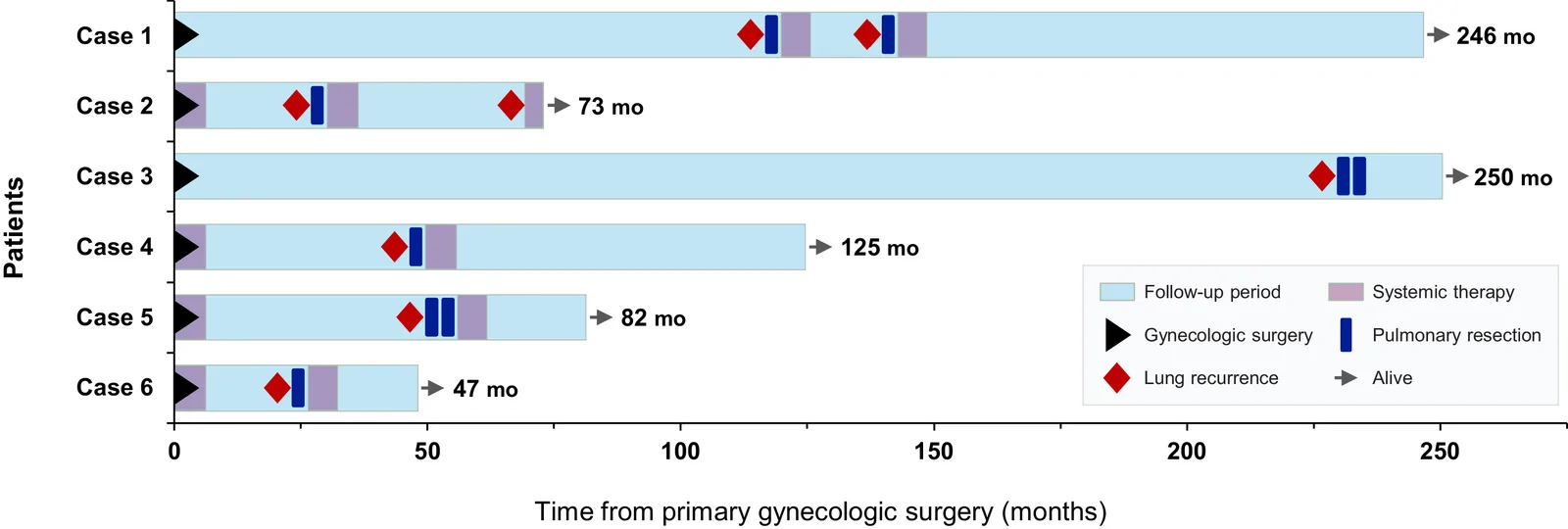
Clinical course of patients with lung-confined recurrent endometrial cancer who underwent pulmonary metastasectomy Each horizontal line represents the clinical course of an individual patient from primary gynecologic surgery to the last follow-up. Symbols indicate the timing of primary gynecologic surgery, adjuvant chemotherapy, pulmonary recurrence, and pulmonary metastasectomy. Follow-up duration is shown in months. Numbers at the end of each line indicate the total duration of follow-up.

Kawanaka. Long-term oncologic outcomes of pulmonary metastasectomy for lung-confined recurrent endometrial cancer. Am J Obstet Gynecol 2026.

Although Case 1 developed a subsequent contralateral lung recurrence 22 months after the initial pulmonary metastasectomy, the recurrent lesion was completely resected by repeat thoracoscopic pulmonary metastasectomy, and the patient remains disease-free more than 20 years after primary treatment. Case 2 underwent complete thoracoscopic resection for the first pulmonary recurrence but subsequently developed multiple pleural metastases 45 months after the initial pulmonary metastasectomy. Because the recurrent disease was no longer amenable to complete surgical resection, the patient is currently receiving paclitaxel–carboplatin plus the immune checkpoint inhibitor durvalumab. In addition, Cases 3 and 5 presented with synchronous bilateral pulmonary metastases and underwent staged bilateral thoracoscopic resections within 1 month, achieving complete resection. Despite bilateral pulmonary involvement, both patients have remained disease-free for prolonged periods following pulmonary metastasectomy.

At the last follow-up, 5 patients had no evidence of disease, whereas one remained alive with disease.

### Characteristics of the first pulmonary recurrence

Characteristics of the first pulmonary recurrence are summarized in Table 2. The median size of the largest pulmonary metastatic lesion was 20.5 mm (range, 9–35 mm). No patient had evidence of extrapulmonary disease at the time of recurrence. Four patients had unilateral pulmonary metastases, whereas 2 presented with bilateral pulmonary metastases. All patients underwent complete (R0) pulmonary resection. When postoperative systemic therapy was administered, it consisted exclusively of conventional cytotoxic chemotherapy (paclitaxel–carboplatin or doxorubicin–cisplatin). Because complete resection was achieved in all patients, no patient required immune checkpoint inhibitors or molecularly targeted therapy after the initial pulmonary metastasectomy. Representative radiological, gross, and histopathological findings of the first pulmonary recurrence are shown in Figure 2.

**Table 2:** Characteristics and surgical management of the first pulmonary recurrence in patients with lung-confined recurrent endometrial cancer.

Case	No. of pulmonary metastases (R/L)	Size of largest pulmonary metastasis, mm	Laterality	Extrapulmonary disease	Pulmonary resection		Postoperative chemotherapy	Subsequent recurrence	Disease status
					Procedure	Resection status			
1	0/1	35	Unilateral (L)	Absent	Lt. upper lobectomy+Lt. S6 segmentectomy	R0	AP (6 cycles)	Yes	NED
2	1/0	12	Unilateral (R)	Absent	Rt. middle lobectomy	R0	TC (6 cycles)	Yes	AWD
3	1/1	25	Bilateral	Absent	Lt. lower lobe wedge resection+Rt. upper lobectomy+Rt. LND	R0	No	No	NED
4	0/1	16	Unilateral (L)	Absent	Lt. upper lobe wedge resection	R0	TC (6 cycles)	No	NED
5	1/1	9	Bilateral	Absent	Lt. upper lobe wedge resection+Rt. S2 segmentectomy	R0	TC (6 cycles)	No	NED
6	0/2	29	Unilateral (L)	Absent	Lt. lower lobectomy+Lt. S3 segmentectomy	R0	TC (6 cycles)	No	NED

*AP*, doxorubicin plus cisplatin; *AWD*, alive with disease; *L*, left; *LND*, lymph node dissection; *NED*, no evidence of disease; *R*, right;

*TC*, paclitaxel plus carboplatin.

**Figure 2:**
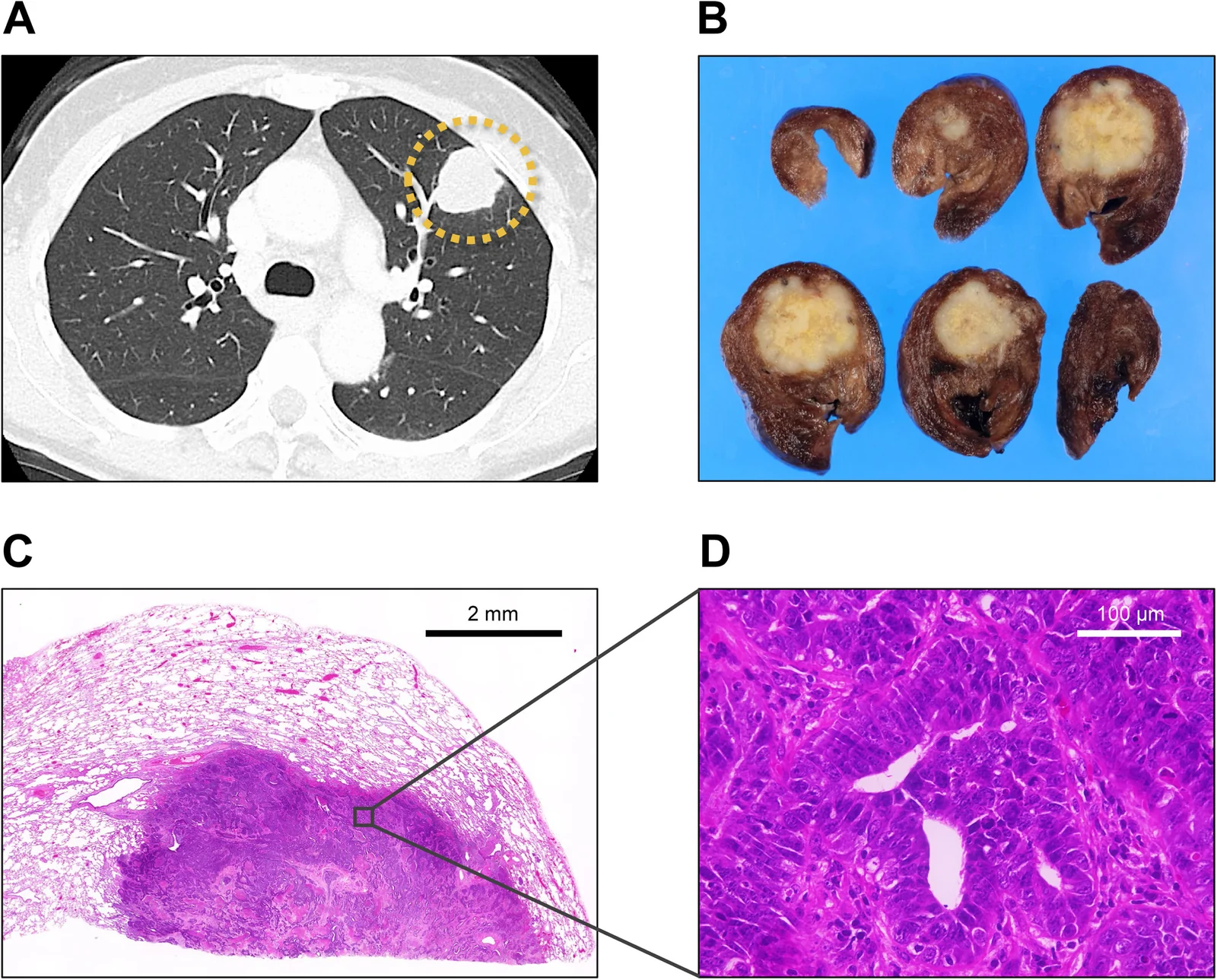
Representative radiological, gross, and histopathological findings of the first pulmonary recurrence A, Chest computed tomography demonstrating a pulmonary nodule in the left upper lobe (dashed circle). B, Gross appearance of the resected pulmonary specimen showing a well-circumscribed whitish tumor. C, Low-power histopathological view of the pulmonary lesion demonstrating metastatic endometrioid carcinoma. D, High-power histopathological view demonstrating the glandular architecture of metastatic endometrioid carcinoma.

Kawanaka. Long-term oncologic outcomes of pulmonary metastasectomy for lung-confined recurrent endometrial cancer. Am J Obstet Gynecol 2026.

### Perioperative outcomes of the initial pulmonary metastasectomy

Perioperative outcomes of the initial pulmonary metastasectomy are summarized in Table 3. The median operative time was 78 minutes (range, 56–190 minutes), and the median estimated blood loss was 0 mL (range, 0–5 mL). No patient required intraoperative blood transfusion. One patient developed postoperative atelectasis, which resolved promptly with conservative management. No other surgery-related adverse events were observed. The median postoperative hospital stay was 9 days (range, 7–13 days). Overall, thoracoscopic pulmonary metastasectomy demonstrated a favorable perioperative safety profile with minimal morbidity.

**Table 3:** Surgical outcomes of the initial pulmonary metastasectomy.

	Total (N = 6)	
Operative findings		
Median operative time, min (range)	78 (56–190)	
Median blood loss, mL (range)	0 (0–5)	
Blood transfusion, n (%)	0 (0.0)	
Surgery-related adverse events	All grades	Grade 3 or 4
Prolonged air leak, n (%)	0 (0.0)	0 (0.0)
Postoperative pneumonia, n (%)	0 (0.0)	0 (0.0)
Atelectasis, n (%)	1 (16.7)	0 (0.0)
ARDS/acute lung injury, n (%)	0 (0.0)	0 (0.0)
Empyema, n (%)	0 (0.0)	0 (0.0)
Postoperative bleeding, n (%)	0 (0.0)	0 (0.0)
Surgical site infection, n (%)	0 (0.0)	0 (0.0)
Postoperative recovery		
Median length of hospital stay, days (range)	9 (7-13)	

*ARDS*, acute respiratory distress syndrome.

### Oncologic outcomes

Kaplan–Meier curves of oncologic outcomes are presented in Figure 3. The Kaplan–Meier estimated median PFS from primary surgery was 44.0 months (Figure 3, A). Following the first pulmonary metastasectomy, 2 patients experienced subsequent recurrence, and the Kaplan–Meier estimated median PM-PFS was 45.0 months (Figure 3, B). One patient underwent repeat pulmonary metastasectomy and remains disease-free, whereas the other developed unresectable recurrence and is currently receiving systemic therapy. All 6 patients were alive at the last follow-up; consequently, PRS and OS remained 100% throughout the observation period (Figure 3, C and D).

**Figure 3:**
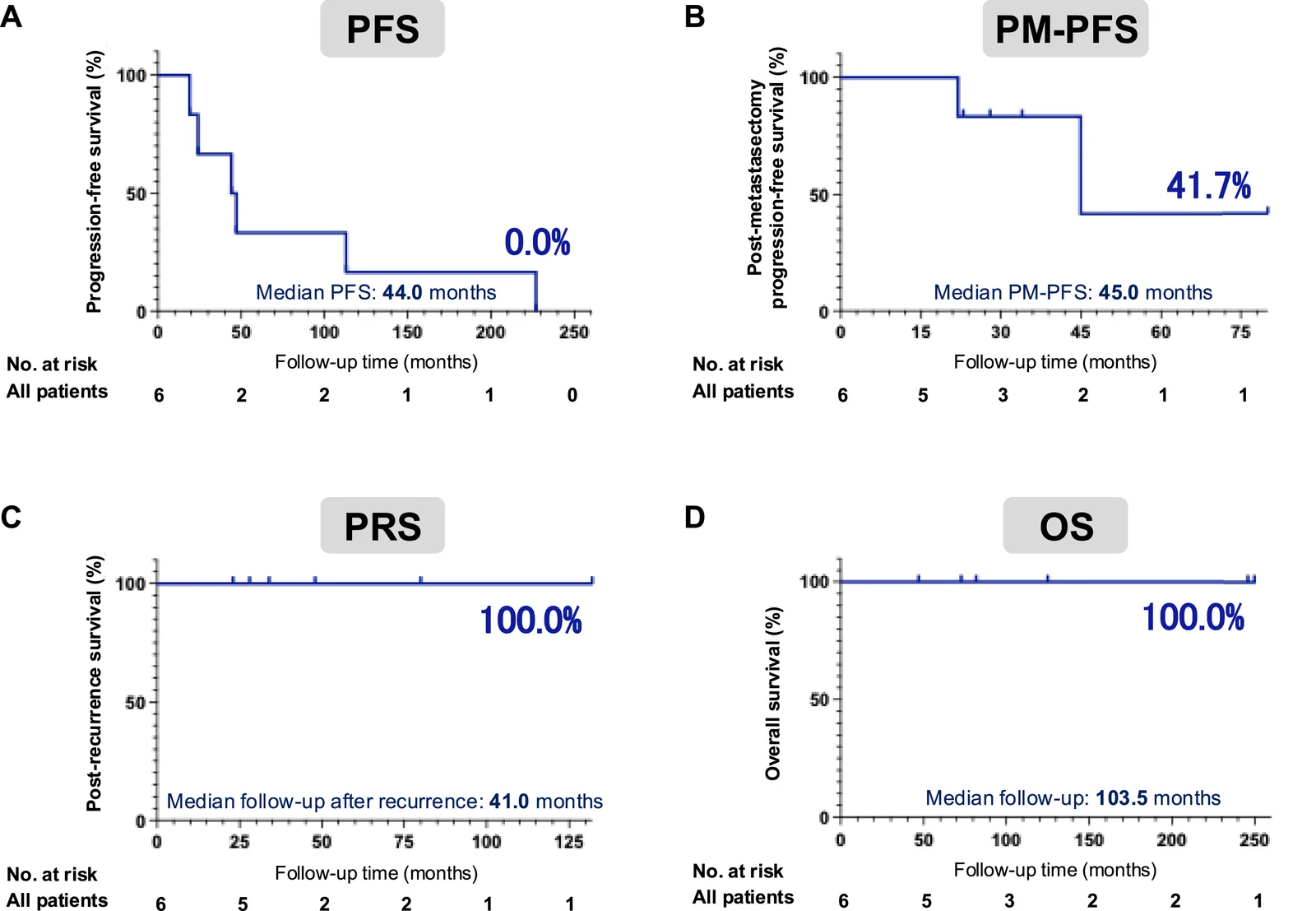
Kaplan–Meier estimates of long-term oncologic outcomes in patients undergoing pulmonary metastasectomy for lung-confined recurrent endometrial cancer A, PFS from primary surgery. B, PM-PFS from the first pulmonary metastasectomy. C, PRS from the first pulmonary recurrence. D, OS from primary surgery. Tick marks indicate censored observations.

Kawanaka. Long-term oncologic outcomes of pulmonary metastasectomy for lung-confined recurrent endometrial cancer. Am J Obstet Gynecol 2026.

## Discussion

The present study demonstrates that carefully selected patients with lung-confined recurrent endometrial cancer can achieve remarkably favorable long-term oncologic outcomes following pulmonary metastasectomy. Despite recurrence after primary treatment, all 6 patients remained alive throughout follow-up, and 5 achieved durable disease-free status. Notably, these outcomes were achieved predominantly before immune checkpoint inhibitors and molecularly targeted therapies became part of routine clinical practice, indicating that durable long-term disease control was achievable with complete surgical resection in appropriately selected patients. These findings have important implications for the contemporary management of recurrent endometrial cancer.

Previous studies have reported favorable outcomes after pulmonary metastasectomy in selected patients with recurrent endometrial cancer.^8^^,^^12^^,^^15^^,^^16^ However, few have specifically focused on lung-confined recurrent endometrial cancer or provided sufficiently long follow-up to assess the durability of disease control. In contrast, the present study exclusively evaluated patients with lung-confined recurrent endometrial cancer who underwent complete pulmonary metastasectomy and were followed for up to 250 months, representing one of the longest follow-up periods reported to date. Importantly, repeat pulmonary metastasectomy remained feasible in a patient who developed subsequent lung-confined recurrence, providing additional durable disease control without compromising long-term survival. Moreover, the 2 patients with synchronous bilateral pulmonary metastases achieved durable disease-free survival following complete staged bilateral resection, suggesting that bilateral pulmonary involvement alone may not preclude long-term disease control when complete resection is feasible. Of note, these favorable oncologic outcomes were achieved with minimal perioperative morbidity, highlighting the feasibility of thoracoscopic pulmonary metastasectomy with limited surgical burden in appropriately selected patients. These findings further support the incorporation of pulmonary metastasectomy into the multidisciplinary management of appropriately selected patients with lung-confined, nondiffuse pulmonary recurrence.

Intriguingly, the findings of the present study challenge the conventional perception that pulmonary recurrence in endometrial cancer invariably represents disseminated systemic disease primarily managed with systemic therapy.^5^ Instead, the present results suggest that lung-confined recurrent endometrial cancer may represent a clinically distinct pattern of recurrence in which pulmonary metastasectomy can provide substantial long-term benefit. The exceptionally prolonged disease-free intervals, the persistent absence of extrapulmonary disease, the feasibility of repeat pulmonary metastasectomy, and the durable long-term survival observed in our cohort collectively support this interpretation.

These clinical observations further raise the possibility that a subset of endometrial cancers possesses a preferential metastatic tropism^17^^,^^18^ for the lungs, resulting in recurrence confined exclusively to the lungs rather than widespread systemic dissemination. Although the biological mechanisms underlying this organ-specific metastatic behavior remain poorly understood,^19^^,^^20^ our findings suggest that lung-confined recurrent endometrial cancer represents a biologically and clinically distinct subset of recurrent disease.^7^ Recognition of this recurrence pattern may facilitate more individualized multidisciplinary management and help prevent potentially resectable disease from being managed with systemic therapy alone.

This study has several limitations. First, it was a retrospective analysis conducted at a single institution with a limited number of patients, which may have introduced selection bias. Second, only patients who were considered suitable candidates for complete pulmonary metastasectomy were included, limiting the generalizability of the findings. Finally, molecular profiling was not uniformly available, precluding exploration of the biological mechanisms underlying the apparent lung-specific metastatic tropism. Nevertheless, the exceptionally long follow-up period, extending beyond 20 years in 2 patients, together with the consistent long-term oncologic outcomes observed in this carefully selected cohort, provides clinically meaningful evidence supporting pulmonary metastasectomy as a treatment option for lung-confined recurrent endometrial cancer.

In conclusion, lung-confined recurrence in endometrial cancer should not invariably be regarded as a manifestation of disseminated systemic disease. In highly selected patients with lung-confined recurrent endometrial cancer, pulmonary metastasectomy can achieve durable long-term disease control and should be considered an integral component of multidisciplinary management. Recognition of lung-confined recurrent endometrial cancer as a biologically and clinically distinct pattern of recurrence may contribute to a shift in the therapeutic paradigm from routine systemic therapy toward a more individualized multidisciplinary approach for carefully selected patients with recurrent endometrial cancer.

## Informed consent

Written informed consent was obtained from all patients in accordance with institutional guidelines.

## CRediT authorship contribution statement

**Kawanaka:** Data curation, Formal analysis, Investigation, Writing – original draft. **Akiho Nishimura:** Formal analysis, Investigation, Methodology, Validation, Writing – review & editing. **Takeshi Motohara:** Conceptualization, Data curation, Formal analysis, Funding acquisition, Investigation, Project administration, Supervision, Visualization, Writing – original draft, Writing – review & editing.
